# An acute pruritic eruption of the trunk in an older adult

**DOI:** 10.1016/j.jdcr.2026.02.040

**Published:** 2026-02-26

**Authors:** Simona Kogan, Joseph Dyer, Sumeet Bhardwaj

**Affiliations:** a Philadelphia College of Osteopathic Medicine, Philadelphia, Pennsylvania; b Philadelphia College of Osteopathic Medicine, Suwanee, Georgia; c Kansas City University College of Osteopathic Medicine, Kansas City, Missouri

**Keywords:** lentinan, shiitake flagellate dermatitis, shiitake mushroom, toxicoderm

## Case description

**Fig 1:**
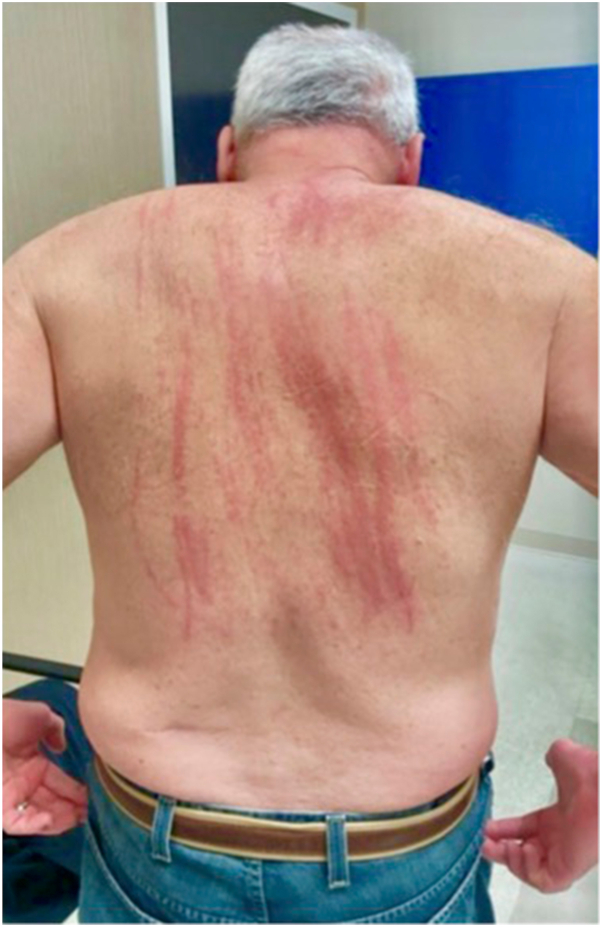
Erythematous Plaques in Streak-like distribution throughout the trunk.

A 72-year-old man presented to the dermatology clinic with intense pruritus involving the trunk and periumbilical region. Physical examination demonstrated erythematous, edematous, linear plaques, most prominent on the left mid–upper back and periumbilical abdomen. The lesions were non-vesicular, non-scarring, and evanescent, with no mucosal involvement or systemic symptoms (Fig 1).

The patient reported ingestion of a large quantity of raw shiitake mushrooms in a salad, with onset of pruritus approximately 48 hours after ingestion. Symptoms progressed and reached peak severity within 2 days. He denied recent illness, new medications, supplements, topical exposures, or a personal or family history of atopy. Symptoms improved following an in-office, single intramuscular injection combination corticosteroid injection consisting of methylprednisolone acetate 40 mg and triamcinolone acetonide 10 mg. He was additionally prescrubed topical triamcinolone acetonide 0.1% cream twice daily, to be applied two weeks per month until resolution.

**Question: What is the name of the toxin causing the diagnosis?**

**A.** Bleomycin
**B.** Lentinan
**C.** Physalis venom
**D.** Furocoumarins

## Discussion

Shiitake flagellate dermatitis is a distinctive toxicoderm classically triggered by ingestion of raw or undercooked shiitake mushrooms. This case demonstrates the typical presentation of intensely pruritic, linear erythematous plaques appearing 24 to 72 hours after exposure, with rapid improvement following corticosteroid therapy and avoidance of re-exposure.^1^ The strong temporal association, characteristic morphology, and self-limited course support a toxin-mediated process rather than a primary allergic reaction.^2^

Current evidence favors lentinan, a heat-labile β-glucan polysaccharide, as the principal causative agent.^3^ The dose-dependent relationship between shiitake consumption and disease severity, along with the marked reduction in cases after adequate cooking, further supports lentinan toxicity as the underlying mechanism.^3^ While hypersensitivity reactions may contribute to select individuals, they are unlikely to be the primary driver.

At higher concentrations, lentinan appears to shift from anti-inflammatory to pro-inflammatory immune modulation. It can act through TLR4 to enhance NF-κB signaling, increase pro-inflammatory cytokine transcription, and prime the NLRP3 inflammasome.^3^^,^^4^ Subsequent minor cutaneous trauma, such as scratching or friction, may provide the second signal for inflammasome activation, resulting in localized cytokine release and the characteristic flagellate eruption.^2^ This proposed mechanism helps explain the delayed onset, linear distribution, and intense pruritus seen in shiitake flagellate dermatitis.

## Conflicts of interest

None disclosed.
